# *CYP1A2* – a novel genetic marker for early aromatase inhibitor response in the treatment of breast cancer patients

**DOI:** 10.1186/s12885-016-2284-3

**Published:** 2016-03-31

**Authors:** Maria Simonsson, Srinivas Veerla, Andrea Markkula, Carsten Rose, Christian Ingvar, Helena Jernström

**Affiliations:** Department of Clinical Sciences, Lund, Division of Oncology and Pathology, Lund University, Lund, Sweden; CREATE Health and Department of Immunotechnology, Lund University, Medicon Village, Lund, Sweden; Department of Clinical Sciences, Lund, Division of Surgery, Lund University and Skåne University Hospital, Lund, Sweden

**Keywords:** Breast cancer, *CYP1A2*, *CYP19A1*, *AhR*, Polymorphisms, Treatment response, Aromatase inhibitor

## Abstract

**Background:**

Endocrine resistance is a major obstacle to optimal treatment effect in breast cancer. Some genetic markers have been proposed to predict response to aromatase inhibitors (AIs) but the data is insufficient. The aim of the study was to find new genetic treatment predictive markers of AIs.

**Methods:**

The ongoing population-based BC-blood study in Lund, Sweden includes women with primary breast cancer. This paper is based on AI-treated patients with estrogen receptor positive tumors who underwent breast cancer surgery in 2002–2008. First, an exploratory analysis of 1931 SNPs in 227 genes involved in absorption, distribution, metabolism, and elimination of multiple medications, using DMET™ chips, was conducted in a subset of the cohort with last follow-up in December 31^st^ 2011 (13 cases, 11 controls). Second, selected SNPs from the first analysis were re-analyzed concerning risk for early breast cancer events in the extended cohort of 201 AI-treated with last follow-up in June 30^th^ 2014. Clinical data were obtained from medical records and population registries.

**Results:**

Only *CYP1A2* rs762551 C-allele was significantly associated with increased risk for early events in the 24 patients (*P* = 0.0007) and in the extended cohort, adjusted Hazard ratio (HR) 2.22 (95 % CI 1.03–4.80). However, the main prognostic impact was found within five years, adjusted HR 7.88 (95 % CI 2.13–29.19). The impact of the *CYP1A2* rs762551 C-allele was modified by a functional polymorphism in the regulator gene *AhR* Arg554Lys (G > A). Compared to patients who were homozygous for the major allele in both genes (*CYP1A2* A/A and *AhR* G/G), a 9-fold risk for early events was found in patients who had at least one minor allele in both genes, adjusted HR 8.95 (95 % CI 2.55–31.35), whereas patients with at least one minor allele in either but not both genes had a 3-fold risk for early events, adjusted HR 2.81 (95 % CI 1.07–7.33). The impact of *CYP1A2* rs762551 C-allele was also modified by the *CYP19A1* rs4646 C/C, adjusted HR 3.39 (95 % CI 1.60–7.16) for this combination. This association was strongest within the first five years, adjusted HR 10.42 (95 % CI 3.45–31.51).

**Conclusion:**

*CYP1A2* rs762551 was identified as a new potential predictive marker for early breast cancer events in AI-treated breast cancer patients. Moreover, combined genotypes of *CYP1A2* rs762551 and *CYP19A1* rs4646 or *AhR* Arg554Lys could further improve prediction of early AI-treatment response. If confirmed, these results may provide a way to more personalized medicine.

## Background

Breast cancer is one of the leading causes of cancer morbidity and mortality among women worldwide [1]. The majority of breast cancer patients have tumors that express hormone receptors [2, 3] and can thus be offered endocrine therapy such as tamoxifen and aromatase inhibitors (AIs). However, endocrine resistance is a major obstacle to optimal treatment effect [4]. Several genetic markers for tamoxifen response have been proposed, although no consensus has yet been reached [5–11]. For AIs, data on genetic markers are sparse [10–13]. The response rates to AIs vary between 35 and 70 % in the neoadjuvant setting [4, 14, 15] and may be lower in advanced disease [16]. By identifying mechanisms of resistance as well as treatment predictive factors, patients may be offered more effective personalized medicine and be spared side-effects of ineffective treatment [17].

Only a few studies have investigated the association between polymorphisms in Cytochrome P450 (CYP) *CYP19A1* (aromatase) and disease-free survival in breast cancer [10, 18, 19]. There are currently only a few studies published with a proposed polymorphism for predicting AI response in the adjuvant setting, and these have contradictory results [11, 13]. Some studies have investigated the impact of *CYP19A1* polymorphisms on treatment response in the metastatic- [20] and in the neoadjuvant settings [21, 22]. However, the results have been inconsistent. Therefore, it is currently unknown whether single nucleotide polymorphisms (SNPs) in *CYP19A1* are associated with a risk of early events in patients treated with AI as first line treatment.

The formation and metabolism of estrogens in the steroidal sex hormone metabolism is complex and involves several enzymes. In addition to CYP19A1, some examples include CYP1A1, CYP1A2, COMT, and CYP3A4 [23]. Several of these enzymes are also involved in the metabolism of AIs [24, 25]. Furthermore, AIs interfere with some of these enzymes; letrozole has been shown to inhibit CYP2A6 and CYP2C19 in vitro [26], anastrozole has been shown to inhibit CYP1A2, CYP2C9, and CYP3A in vitro [27], and exemestane has been shown to be metabolized by CYP4A11 and CYP1A1/2 in vitro [28]. Polymorphisms in the corresponding genes may be a mechanism behind primary (*de novo*) resistance of AI as estrogens are known risk factors for recurrence of breast cancer and the enzymes that metabolize estrogens are tightly linked to AI metabolism. Two of these genes involved in estrogen metabolism, *CYP1A1* and *CYP1A2*, share a common promoter [29] and are under regulatory control of the aryl hydrocarbon receptor (AhR) [30]. These genes may therefore be of interest to study in relation to AI response.

To find new markers beyond the candidate genes for AI resistance, it might be useful to expand the search to other known genes involved in Absorption, Distribution, Metabolism, and Elimination (ADME-related genes). High-throughput, drug metabolism enzymes and transporters (DMET™) chips genotype several SNPs at the same time [31]. The Affymetrix DMET Plus Premier Pack includes 1931 SNPs in 227 genes in ADME-related genes on a single array. We hypothesized that SNPs in the aromatase gene *CYP19A1* and SNPs in other genes for drug and estrogen metabolism may be used as treatment predictive markers for adjuvant treatment with AI in primary breast cancer patients. The aim of the study was: 1) to perform an exploratory analysis using the DMET™ chip to find new treatment predictive markers in a subset of the cohort and 2) to examine these potential markers with a special focus on *CYP19A1* in relation to a risk for early events in the extended cohort of AI-treated breast cancer patients.

## Methods

### Study population

Women diagnosed with a primary breast cancer at the Skåne University Hospital in Lund, Sweden were invited preoperatively to participate in an ongoing prospective population-based cohort—the BC-blood study. Patients with a prior history of another cancer diagnosis within the last ten years were not enrolled. The overall aims of the BC-blood study are to elucidate factors that may have prognostic or predictive value. This paper is based on data collected from 634 primary breast cancer patients between October 2002 and October 2008. Patients were followed from inclusion to the first breast cancer event or distant metastasis, respectively, and patients without events were censored at the last follow-up or death prior to July 1^st^ 2014. As previously described, the follow-up rates of the patients were high [32]. During the time the cohort was compiled, 1090 patients went through breast cancer surgery and approximately 58 % of these patients were included [33]. A lack of research nurses explains most of the patients who were missed and approximately 5 % of the patients were missed due to unverified diagnosis at the time of surgery. Written informed consent was obtained from all patients, and the study was approved by the ethics committee of Lund University (Dnr LU75-02, LU37-08, and LU658-09).

Breast cancer events included ipsilateral, contralateral, axillary lymph node, and distant metastases. Information concerning breast cancer events was obtained from patient charts, pathology reports, and the Regional Tumor Registry. The date of death was obtained from the Swedish Population Registry. The first breast cancer event of any type was considered the primary endpoint, and distant metastasis was considered a secondary endpoint. Breast cancer treatment was prescribed according to the standard of care at Skåne University hospital. Information regarding the type of adjuvant treatment was collected from patient charts and questionnaires. Treatment data were registered up to the last follow-up prior to any event. Data on tumor size, histological type and grade, and number of involved axillary lymph nodes were obtained from each patient’s pathology report. The tumors were analyzed at the Department of Pathology at Lund University Hospital. Estrogen receptor (ER) and progesterone receptor (PgR) status were determined as previously described [5, 34]. The patients completed questionnaires preoperatively and at multiple times postoperatively. The questionnaires included questions such as reproductive history, use of exogenous hormones, smoking history, and any medications used during the past week as previously described [35]. During the preoperative visit, a research nurse collected blood samples for genotyping. The blood was collected and centrifuged and the samples were frozen at −80 ° C within two hours.

### Genotyping

Genomic deoxyribonucleic acid (DNA) was extracted from the patients’ leukocyte portion of frozen peripheral blood using the Wizard Genomic DNA Purification Kit (Promega, Madison, USA) or Quickgene-610 L and Quickgene-810 (Fujifilm life science, Science imaging AB, Scandinavia). The samples were then genotyped using the DMET™ (Drug Metabolizing Enzymes and Transporters) Plus Premier Pack, which is a microarray assay developed by Affymetrix (Santa Clara, CA, USA), according to the manufacturer’s instructions. The DMET™ experimental analysis was performed at SCIBLU Genomics at Lund University.

Genotyping was also performed at the Region Skåne Competence Centre (RSKC Malmö), Skåne University Hospital, Malmö, Sweden. The *CYP19A1* SNPs rs700518, rs4646, Aro1 (rs4775936), Aro2 (rs10459592), and two functional AhR SNPs Arg554Lys (rs2066853) and Val570Ile (rs4986826) were analyzed with matrix-assisted laser desorption/ionization time-of-flight mass spectrometry on a Sequenom MassARRAY® platform (Sequenom, San Diego, CA, USA), using iPLEX reagents according to the manufacturers’ protocol. The Sequenom MassARRAY® software was used for multiplex SNP analysis design. The rs700518 SNP was not successfully genotyped. The rs10046 SNP and the CYP1A2*1 F (rs762551) SNP were genotyped using a Taqman SNP allelic discrimination assay in 384-well format on an ABI PRISM 7900 Sequence Detection System (Applied Biosystems, Foster City, CA, USA). Over 10 % of the samples were run in duplicates with a concordance of 100 %.

There were 19 patients not successfully genotyped for the CYP1A2*1 F (rs762551) SNP using TaqMan. For 16 of these patients, rs762551 genotypes were available from DNA sequencing from a previous study [18]. The concordance rate between the two methods was 99.8 %. Haplotypes of *CYP19A1* were constructed by cross-tabulation of the genotypes of the *CYP19A1* SNPs. This resulted in nine haplotypes. Linkage disequilibrium (LD) was observed between rs4646 and rs10046 (*r* = 0.68), and between rs10046 and Aro1 (*r* = 0.90), as well as between Aro1 and Aro2 (*r* = 0.79). Therefore, the 14 missing genotypes for rs10046 could be imputed. The minor allele was defined according to the Database of Single Nucleotide Polymorphisms (dbSNP) [36].

### Data analyses

The analyses of data from the DMET™ chip were performed using the DMET™ console software. The samples with QC call rates ≥99 % were considered for further analyses. The analysis included 13 patients with breast cancer events who had been treated with AI but not with chemotherapy prior to the event by December 31^st^ 2011 (*n* = 13). The controls were 11 AI-treated patients without chemotherapy or tamoxifen and without recurrence who had a follow-up time of at least five years with last follow-up December 31^st^ 2011. Fisher’s exact test was used, and to make allowances for multiple testing, a *P-*value < 0.005 was considered significant. This *P*-value allows for 0.5 % of the findings to be false positive.

**Fig. 1:**
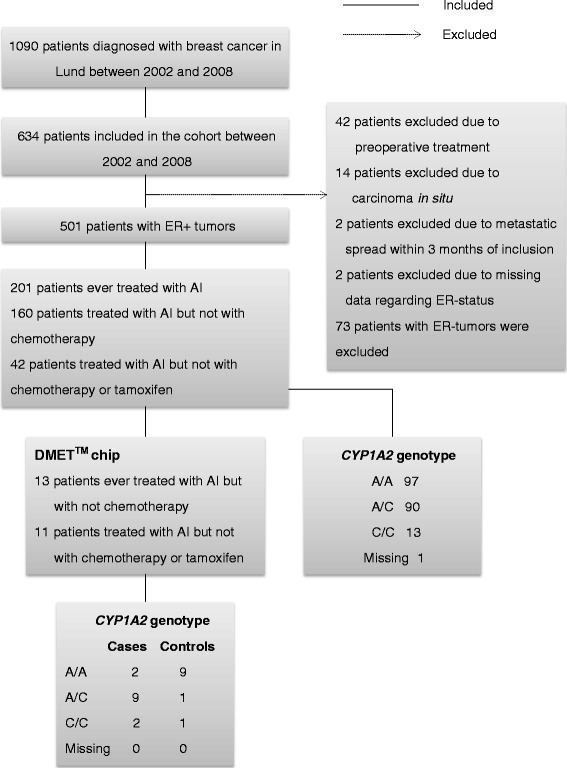
Flowchart illustrating patients included and excluded in the different analyses

Statistical survival analyses of the extended cohort were performed with IBM SPSS Statistics, version 19.0 (IBM Corp. Armonk, NY, USA). A flowchart of patients included in the final survival analyses is presented in Fig. 1. After exclusion, 201 AI-treated patients were included in the analyses. In total, 32 patients were diagnosed with some type of breast cancer event during the 11-year follow-up period. Of these, 22 presented with distant metastases. A Kaplan-Meier LogRank test was used for univariable analyses of the risk of early events in relation to the different genotypes, haplotypes, and diplotypes of the SNPs. Since few patients had an invasive tumor size ≥51 mm or muscular or skin involvement, these patients were combined with the patients with invasive tumor sizes between 21 and 50 mm in the multivariable analyses. Regular smokers and occasional smokers were classified as current smokers. Cox regression was used to calculate Hazard Ratios (HRs) in relation to the SNPs after adjusting for age (linear), invasive tumor size (<21 mm versus ≥21 mm or skin or muscular involvement independent of size), any axillary lymph node involvement (yes/no), histological grade III (yes/no), preoperative smoking status (yes/no), body mass index (BMI) ≥25 kg/m^2^ (yes/no) radiation therapy (yes/no), chemotherapy (yes/no), and tamoxifen therapy (yes/no). A *P*-value < 0.05 was considered significant. All *P*-values were two-tailed. Nominal *P*-values are presented without adjustments for multiple testing since this is an exploratory study [37]. A prior power calculation assuming a study with 200 patients; 50 % of the patients had a major allele and an accrual interval of 6 years with additional follow-up after the accrual interval of 4 years, showed that the study could detect true HRs of failure for patients homozygous for the major allele relative to patients with variant alleles of 0.603 or 1.824 with 80 % power and a type 1 error probability of 0.05 [38]. The study is based on the REMARK (Reporting Recommendations for Tumor Marker Prognostic Studies) criteria [39].

## Results

### Patient characteristics, tumor characteristics, and AI treatment

Patient and tumor characteristics of the patients are presented in Tables 1 and 2, respectively. There were no substantial differences in the characteristics between the AI-treated patients in the extended cohort and the patients analyzed with the DMET™ chip other than age and height. The distribution of AIs was as follows: anastrozole 67.2 %, letrozole 26.4 %, exemestane 5.0 %, anastrozole and letrozole in sequence 1.0 %, and AI type missing 0.5 %.

**Table 1:** Patient characteristics of the AI-treated patients with ER+ tumors included in the DMET™ chip analysis and the extended cohort

	Patients included in the analysis of the DMET™ chip		Patients in the extended cohort included in the survival analyses	
	n = 24		n = 201	
	Median (IQR) or %	Missing	Median (IQR) or %	Missing
Age at diagnosis, yrs	67.7 (60.0–72.7)	0	60.9 (54.4–66.4)	0
Weight, kgs	70.0 (61.7–82.10)	0	70.0 (64.0–79.0)	2
Height, m	1.64 (1.58–1.68)	0	1.66 (1.62–1.70)	0
BMI, kgs/m^2^	26.6 (23.6–30.6)	0	25.2 (23.2–28.8)	2
Age at menarche, yrs	14.0 (13.0–14.0)	0	13.0 (12.0–14.0)	0
Parous, %	87.5 %	0	85.1 %	0
Age at first full-term pregnancy, yrs^a^	23.5 (21.0–26.8)	3	24.0 (22.0–27.0)	31
Ever use of oral contraceptives, %	62.5 %	0	70.6 %	0
Ever use of hormone therapy, %	45.8 %	0	54.0 %	1
Current smoker prior to surgery, %	20.8 %	0	17.4 %	0
Alcohol abstainers	16.9 %	0	10.0 %	1
Preoperative daily coffee consumption 2 + cups/day	75.0 %	0	83.5 %	1

^**a**^Of the parous patients

**Table 2:** Tumor characteristics of the AI-treated patients with ER+ tumors included in the DMET™ chip analysis and the extended cohort

	Patients included in the analysis of the DMET™ chip		Patients in the extended cohort included in the survival analyses	
	n = 24		n = 201	
	Number (%)	Missing	Number (%)	Missing
Invasive tumor size, mm (stage)		0		0
≤20 (pT1)	15 (62.5 %)		128 (63.7 %)	
21–50 (pT2)	8 (33.3 %)		69 (34.3 %)	
51–(pT3)	1 (4.2 %)		4 (2.0 %)	
Skin or muscular involvement (pT4)	0 (0 %)		0 (0 %)	
≥21 mm or skin or muscular involvement	9 (37.5 %)		73 (36.3 %)	
Axillary node involvement		0		0
0	8 (33.3 %)		55 (27.4 %)	
1–3	9 (37.5 %)		107 (53.2 %)	
4+	7 (29.2 %)		39 (19.4 %)	
Any axillary lymph node	16 (66.7 %)		146 (72.6 %)	
Histological grade		0		0
I	6 (25.0 %)		44 (21.9 %)	
II	16 (66.7 %)		124 (61.7 %)	
III	2 (8.3 %)		33 (16.4 %)	
Hormone receptor status		0		0
ER+	24 (100.0 %)		201 (100.0 %)	
PgR+	20 (83.3 %)		161 (80.1 %)	

### DMET™ analysis and selection of SNPs for further analyses

Of the 1931 SNPs, 1911 were successfully genotyped. Only the CYP1A2*1F rs762551 C-allele was significantly associated with increased risk for early events among the 24 AI-treated patients (*P* = 0.0007). The *CYP1A2* rs762551 was thus elected for analyses in the extended cohort. The *CYP19A1* SNPs were not significantly associated with survival in the analyses of the DMET™ chip in the 24 patients. The first *CYP19A1* SNP, rs700518, appeared in 12^th^ place (*P* = 0.014). However, a special focus was placed on *CYP19A1* in this paper since aromatase is the target of AIs. Therefore, five *CYP19A1* SNPs (rs700518, rs4646, rs10046, Aro1, and Aro2) were also selected for survival analyses in the extended cohort. Moreover, since *AhR* is involved in the regulation of *CYP1A2*, genotyping was also performed for two functional *AhR* SNPs Arg554Lys (rs2066853) and Val570Ile (rs4986826) of which only Arg554Lys was included in the DMET™ chip.

### *CYP1A2* rs762551 in relation to risk for early events in AI-treated patients

**Fig. 2:**
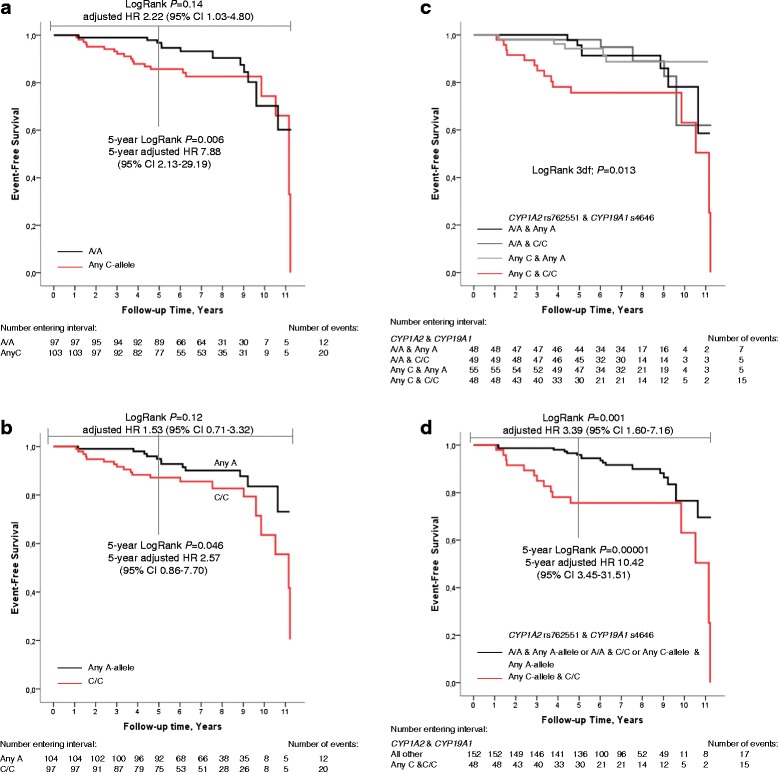
**a**-**d** Kaplan-Meier estimates of event-free survival in relation to *CYP1A2* and *CYP19A1* genotypes in AI-treated breast cancer patients with ER+ tumors are illustrated. LogRank *P*-values are presented for the entire follow-up time. In Fig. 2 **a**, **b**, and **d**, 5-year adjusted HRs are also presented. **a** *CYP1A2* rs762551. The main association between *CYP1A2* rs762551 any C-allele and early events was observed within 5 years of inclusion. **b** *CYP19A1* rs4646. No significant association between *CYP19A1* rs4646 and early events was observed. **c** Combinations of *CYP1A2* rs762551 and *CYP19A1* rs4646 genotype. Patients with any C-allele of *CYP1A2* rs762551 and C/C genotype of rs4646 had a worse prognosis compared to patients with the three other genotype combinations. **d** *CYP1A2* rs762551 any C-allele and rs4646 C/C. A combined variable of any C-allele of *CYP1A2* rs762551 and C/C genotype of rs4646 was created and patients with this combination had a worse prognosis compared to patients with any other genotype. The main association was observed within 5 years of inclusion

The patients were followed for up to 11 years with a median follow-up time of 7.2 years (IQR 5.3–9.2) for patients who were alive and still at risk at the last follow-up. The minor allele frequency (MAF) was 29.0 % for *CYP1A2* rs762551 (C-allele). AI-treated patients with ER+ tumors and any C-allele of *CYP1A2* rs762551 genotype (*n* = 103) had a significantly higher risk for early breast cancer events versus the patients with A/A genotype (Fig. 2a; adjusted HR 2.22 (95 % CI 1.03–4.80). However, the main treatment predictive impact of *CYP1A2* rs762551 was found within five years of inclusion (early events), adjusted HR 7.88 (95 % CI 2.13–29.19).

**Fig. 3:**
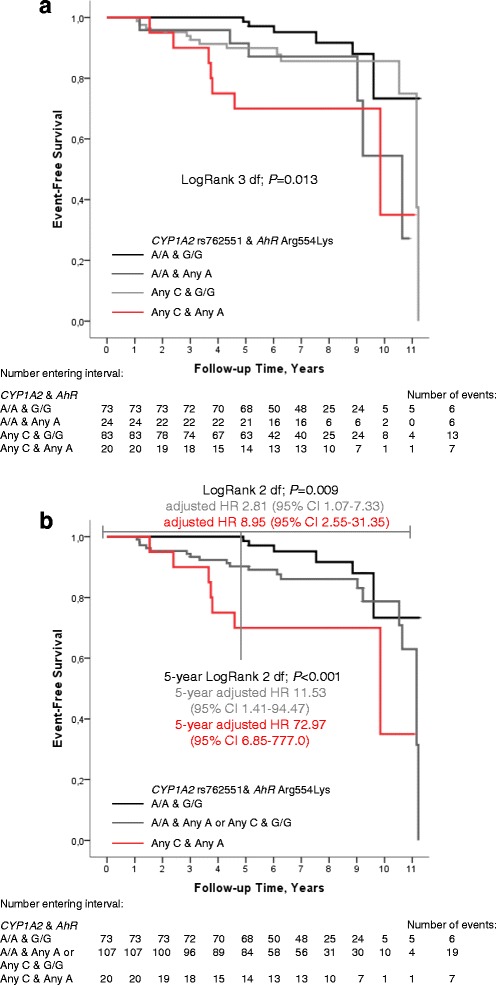
**a** Combinations of *CYP1A2* rs762551 and *Ahr* Arg554Lys genotype. Patients with any C-allele of *CYP1A2* rs762551 and any A-allele of *AhR* Arg554Lys had a worse prognosis followed by patients with at least one minor allele in either but not both genes and the lowest risk was seen in patients with the *CYP1A2* rs762551 A/A genotype combined with the *AhR* Arg554Lys G/G genotype. **b** Three combinations of *CYP1A2* rs762551 and *Ahr* Arg554Lys genotype. Patients who had a CYP1A2 A/A genotype and AhR any A-allele or CYP1A2 any C-allele and AhR G/G genotype were combined to one moderate risk group as the curves were similar in these groups. Patients with *CYP1A2* rs762551 any C-allele and *AhR* Arg554Lys any A-allele had the highest risk for early events, followed by the combined group, compared to patients with *CYP1A2* rs762551 A/A genotype and *AhR* Arg554Lys G/G genotype. Please note that there are fewer patients with longer follow-up times as this is an on-going cohort

When the patients ever treated with chemotherapy were excluded as was done in the DMET™ analysis, 159 patients remained and 26 events occurred until 30^th^ June 2014. Here, the association did not remain significant, adjusted HR 1.97 (95 % CI 0.84–4.59) but a significant impact was found within five years of inclusion, adjusted HR 7.22 (95 % CI 1.49–40.00).

After exclusion of patients ever treated with tamoxifen and/or chemotherapy, only 42 patients remained and 8 events occurred. The association was significant in the univariable model (LogRank *P* = 0.002) and for events within five years (*P* = 0.032). Due to small numbers, no Cox regression was performed. The AI-treated patients with ER+ tumors and any C-allele of *CYP1A2* rs762551 also had a significantly increased risk for early distant metastases overall (LogRank *P* = 0.020), adjusted HR 3.47 (95 % CI 1.26–9.56) and within five years (LogRank *P* = 0.020), adjusted HR 7.80 (95 % CI 1.51–40.32).

### Combination of *CYP1A2* and *AhR*

Genotyping of the *AhR* SNP Val570Ile (rs4986826) was non-informatory since all patients had the G/G genotype. The minor allele frequency for Arg554Lys (rs2066853 A-allele, Lys) was 11.9 %. There was no linkage between the *CYP1A2* rs762551 and *AhR* Arg554Lys genotypes. *AhR* Arg554Lys was not associated with early events in the patients included in the analysis of the DMET^TM^ chip and appeared in 524^th^ place. However, in the extended cohort, patients with any A-allele of the *AhR* Arg554Lys had a significantly higher risk for early events compared to patients with the G/G genotype overall (LogRank *P* = 0.005), adjusted HR 2.61 (95 % CI 1.24–5.50) and within five years (LogRank *P* = 0.013), adjusted HR 3.33 (95 % CI 1.24–8.96).

There was no interaction between the *CYP1A2* rs762551 and *AhR* Arg554Lys. However, a combination of the two SNPs showed multiplicative associations. Patients who had at least one minor allele in both genes, i.e., any *CYP1A2* C-allele and any *AhR* A-allele, had the highest risk for early events followed by patients who had a *CYP1A2* A/A genotype and *AhR* any A-allele or *CYP1A2* any C-allele and *AhR* G/G compared to patients who were homozygous for the major allele in both genes *CYP1A2* A/A and *AhR* G/G (LogRank 3 df; *P* = 0.013), Fig. 3a. Since the curves for patients with *CYP1A2* A/A and *AhR* any A-allele or *CYP1A2* any C-allele and *AhR* G/G overlapped, these genotypes were combined in the multivariable model into one group of patients that were homozygous for the major allele in one but not both genes. Overall, compared to patients who were homozygous for the major allele in both genes, patients who had at least one minor allele in both genes (*n* = 20, 7 events) had a 9-fold risk for early events, adjusted HR 8.95 (95 % CI 2.55–31.35), whereas patients with at least one minor allele in one but not both genes (*n* = 107, 19 events) had a 3-fold risk for early events, adjusted HR 2.81 (95 % CI 1.07–7.33). These results were also seen when the analysis was restricted to the first five years (LogRank 3 df; *P* < 0.001), Fig. 3b.

### *CYP19A1* in relation to risk for early events in AI-treated patients

The *CYP19A1* SNP rs700518 was not successfully genotyped using iPlex and could not be further analyzed. The MAF for rs4646 (A-allele), rs10046 (T-allele), Aro1 (T-allele), and Aro2 (T-allele) were 30.8, 49.3, 47.0 and 40.0 %, respectively. In line with the DMET™ data, the genotypes, haplotypes, and diplotypes of the four *CYP19A1* SNPs were not associated with early events (all adjusted *P-*values > 0.10; see Fig. 2b for rs4646). Excluding the patients ever treated with chemotherapy and/or tamoxifen did not materially change the result. All of the results remained insignificant in relation to risk for distant metastases.

### Combination *of CYP19A1* SNPs and *CYP1A2*

To investigate whether the findings regarding risk for early events and the *CYP1A2* rs762551 SNP were modified by the *CYP19A1* SNPs, stratification according to each genotype of the four *CYP19A1* SNPs was performed. No effect modification was observed between the strata for rs10046, Aro1, Aro2, and *CYP1A2* rs762551. The interaction analyses were non-significant. However, the interaction between *CYP1A2* rs762551 any C-allele and the C/C genotype of *CYP19A1* rs4646 was significant (adjusted *P*_*interaction*_ = 0.022). Any C-allele carriers of *CYP1A2* rs762551 with the C/C genotype of *CYP19A1* rs4646 (*n* = 48, 15 events) had over a 3-fold increased risk of early events versus the rest of the AI-treated patients (Fig. 2c-d; LogRank *P* = 0.001), adjusted HR 3.39 (95 % CI 1.60–7.16). As with *CYP1A2* rs762551 alone, the main treatment predictive impact of was found within five years (LogRank P = 0.00001), adjusted HR 10.42 (95 % CI 3.45–31.51).

## Discussion

The present study investigated the association between SNPs in ADME-related genes and the risk of early breast cancer events in AI-treated patients with primary breast cancer. The main finding was that *CYP1A2* rs762551 was significantly associated with risk of early breast cancer events among AI-treated patients with ER+ tumors, both in the exploratory analysis and in the extended cohort. This suggests that *CYP1A2* rs762551 may be a predictive marker for early AI-response. To the best of our knowledge, this has not been reported before.

The DMET™ chip was selected because the included SNPs are involved in genes of importance for drug metabolism and transportation. This approach increases the chance that a finding is of biological relevance for AI response. The cut-off for the *P*-value in the DMET™ analysis was chosen to allow for identification of potentially new candidate genes while keeping the number of false positive findings low. As this was an exploratory analysis of nearly 2000 SNPs, a Bonferroni correction would have been too stringent and the risk for false negative findings substantial. The *CYP1A2* rs762551 was the only SNP that met the predetermined cut-off and the enzyme is involved in the metabolic pathways of AIs or is inhibited by AIs [25, 27, 28], which increases the chance that the finding may be of biological relevance.

CYP1A2 is a phase I pathway for drug metabolism and elimination [40]. An in vitro study reported a significant role of CYP1A2 in exemestane metabolism [28]. Moreover, CYP1A2 catalyzes the conversion of estradiol to hydroxylated metabolites, primarily 2-hydroxylated estradiol [23], which has been shown to act as a weak or even as an anti-estrogenic substance [41]. In a subset of 59 patients in the current cohort, the *CYP1A2* rs762551 C-allele was associated with a low 2OHE-to-16alphaOHE1 plasma ratio both pre- and post-operatively [42]. However, none of these patients were treated with AIs at the time of blood draw. Since AIs block estrogen formation, it is unlikely that there are measurable estrogen metabolite plasma levels in the 201 AI-treated patients.

The *CYP1A2* rs762551 is located in intron 1 of the *CYP1A2* gene and carries a -163C > A substitution. CYP1A1/2 expression is regulated by the AhR and a number of transcription factors and might be influenced by transcriptional coactivators and corepressors [43]. The A/A genotype of *CYP1A2* rs762551 is highly inducible especially by smoking [44] and coffee consumption [45]. Neither smoking nor coffee consumption accounted for the association between *CYP1A2*, *AhR*, and risk for early events (data not shown). Furthermore, all multivariable models were adjusted for smoking. While the *CYP1A2* rs762551 has been shown to influence inducibility, it has not been shown to significantly alter the gene expression [46]. The results are conflicting as to whether the SNP influences CYP1A2 enzyme activity [43, 46]. Aklillu et al. have performed extensive characterization of *CYP1A2* genotype phenotype correlations [47]. Cell transfection experiments showed that there was no significant difference in the constitutive transcriptional activity depending on the *CYP1A2* rs762551 SNP. Further, electrophoretic mobility shift assay analysis could not identify any specific transcription factor whose binding could be affected by rs762551. However, a xenobiotic response element (XRE) containing an invariant CACGC core sequence, recognized by AhR, is present in *CYP1A2* intron 1 further downstream of the rs762551 site [47]. In the current study, a multiplicative association between having at least one *CYP1A2* rs762551 C-allele and at least one *AhR* Arg554Lys A-allele on the risk for early events in AI-treated patients was observed. Helmig et al. reported that the *AhR* A-allele (Lys) confers lower expression of AhR compared to the G-allele (Arg) [48]. Further, there is cross-talk between AhR and ERα. An animal rat model showed that ligand-activated AhR confers anti-estrogenic effects partly due to lower ERα levels in ductal epithelial cells [49]. CYP1A2 is mainly expressed in liver cells but has also been detected in the ER+ breast cancer MCF-7 cell-line after induction [30]. In the current study, neither *AhR* nor *CYP1A2* were associated with prognosis among the patients with ER+ tumors who had not been treated with AIs but either received tamoxifen or no endocrine treatment (data not shown). This suggests that the association of *AhR* Arg554Lys and *CYP1A2* rs762551 on prognosis may be exclusive for the AI-treated patients where the ER is still open as opposed to tamoxifen-treated patients where the ER is blocked. The cross-talk between AhR and ER signaling may be one mechanism behind these findings. Taken together, this suggests that *AhR* G/G carries may have both lower ERα levels and more effective *CYP1A2* transcription and expression. This may be especially pronounced in patients with the highly inducible *CYP1A2* A/A genotype, since AhR regulates *CYP1A2* expression, thus leading to a lower risk for early events during AI-treatment.

In addition to AhR, other c*is*- or *trans* acting loci may also regulate *CYP1A2* gene expression levels [50]. The CYP1A2 enzyme activity level and gene expression is clustered with CYP2C8, CYP2C9, and CYP3A4 [51]. Further, *CYP1A2* share a common promoter with *CYP1A1* [29]. However, *CYP1A1*, *CYP2C8*, *CYP2C9*, and *CYP3A4* were not significant in the analysis based on the DMET™ chip data, therefore no further analyses was performed here.

While the role of *CYP1A2* rs762551 with respect to breast cancer risk seems weak or non-significant [52], unless coffee consumption was taken into account [34, 53]. A meta-analysis showed that the association between the *AhR* Arg554Lys and breast cancer risk differs between studies of women with different ethnicities, although the overall result was no association [54]. The *CYP1A2* rs762551 is associated with the metabolism of several drugs and also with efficacy and toxicity [43]. Although the mechanism behind the finding of the present study is not fully understood, the current study provides new insight into how the *CYP1A2* rs762551 combined with the functional *AhR* Arg554Lys variant is linked to prognosis in AI-treated breast cancer patients. Moreover, AhR appears to be involved in the regulation of *CYP19A1* both in ovarian and adrenocortical cells via different mechanisms [55]. Further mechanistic and translational studies of the AhR and CYP1A1/2 signaling pathway with respect to *CYP19A1* regulation and AI-response are therefore warranted.

In the present study, a special focus was placed on *CYP19A1* since aromatase is the target of AIs. However, genotypes, haplotypes, and diplotypes of *CYP19A1* SNPs rs4646, rs10046, Aro1, and Aro2 were not significantly associated with a risk of early breast cancer events in the AI-treated patients. These results are in line with the recent study by of Leyland-Jones et al. [11] that investigated *CYP19A1* tumor genotype data in relation to endocrine treatment response in the BIG 1–98 trial, but in contrast to a previous study on genomic *CYP19A1* genotypes regarding the risk of recurrence in AI-treated patients [13]. In the recent abstract by Umamasheran et al., an increased risk for breast cancer recurrence was observed among 191 Indian letrozole-treated T/T carriers of rs4646 [13]. As mentioned in the background, several SNPs in *CYP19A1* have previously been associated with AI response in neoadjuvant and metastatic settings. The rs4646 is among the most frequently studied SNPs. The A-allele has previously been associated with longer time to progression in the metastatic setting [20] but with poor response in the neoadjuvant setting [22]. The differences may be due to ethnicity, different study types, and small study populations. Furthermore, different types of AIs may yield different results. The majority of the patients included in the present study had received anastrozole. A recent study by Lunardi et al. reported no association between plasma estrone concentrations during treatment with letrozole and rs4646 or rs10046 [56]. Since circulating estrone levels may influence the risk for early events in breast cancer, this is in line with the findings of no significant association between these SNPs and early events in the present study.

In the present study, there was a significant interaction between *CYP1A2* rs762551 and *CYP19A1* rs4646 with a high risk for breast cancer events, especially within five years, among patients with any C-allele of *CYP1A2* rs762551 and C/C-carriers of *CYP19A1* rs4646. This subgroup was quite small, and the results need to be interpreted with caution. There was no linkage between *CYP19A1* rs4646 and *AhR* Arg554Lys that could explain the results (data not shown). All findings in the present study warrant validation, preferably within a randomized clinical trial. In such a trial, it would be possible to elucidate whether these genotypes are associated with AI-response. If so, this could guide selection of endocrine treatment for more personalized medicine in the clinical setting.

The Skåne University Hospital in Lund has a catchment area that includes almost 300,000 inhabitants. This study is population-based since patients with breast cancer diagnoses are not referred to other hospitals for surgery. The vast majority of the patients who are diagnosed in Lund are Swedes, but no data on ethnicity was collected. Thus, studies with different study populations are warranted. Since the subset of the cohort that was analyzed with the DMET™ chip also was part of the extended cohort from which they originated, all findings warrant validation in an independent cohort. Further, the follow-up period was relatively short—patients with ER+ tumors tend to relapse later [57]. Therefore, the long-term effects of *CYP1A2* rs762551 or *CYP19A1* rs4646 could not be evaluated. However, the main impact of these SNPs was observed during the 5-year period when endocrine treatment is administered. This suggests that these SNPs may be involved in primary rather than acquired resistance.

## Conclusions

This study identified a new potential AI-treatment predictive marker in *CYP1A2* rs762551 for early breast cancer events, and the results indicate that *CYP1A2* rs762551 in combination with *CYP19A1* rs4646 or *AhR* Arg554Lys may yield even better treatment prediction. The results of the current study indicate that *CYP1A2* rs762551 and the *AhR* signaling pathway merit further study for AI-response. If confirmed, these results may provide a way to more personalized medicine.

## Abbreviations

ADME-related genes: absorption, distribution, metabolism, and elimination-related genes
AhR: aryl hydrocarbon receptor
AI: aromatase inhibitors
BMI: body mass index
CYP: cytochrome P450
DMET: drug metabolism enzymes and transporters
DNA: deoxyribonucleic acid
ER: estrogen receptor
IQR: interquartile range
MAF: minor allele frequency
PgR: progesterone receptor
SNP: single nucleotide polymorphism
WHR: waist-to-hip ratio
XRE: xenobiotic response element
